# The Kager’s fat pad radiological anatomy revised

**DOI:** 10.1007/s00276-020-02552-1

**Published:** 2020-08-19

**Authors:** Paweł Szaro, Mateusz Polaczek, Bogdan Ciszek

**Affiliations:** 1 Department of Radiology, Institute of Clinical Sciences, Sahlgrenska Academy, University of Gothenburg, Göteborgsvägen 31, 431 80 Gothenburg, Sweden; 2 Department of Musculoskeletal Radiology, Sahlgrenska University Hospital, Göteborgsvägen 31, 431 80 Gothenburg, Sweden; 3 Third Department of Lung Diseases and Oncology, National Tuberculosis and Lung Diseases Research Institute, Plocka 26, 01138 Warsaw, Poland; 4 Department of Descriptive and Clinical Anatomy, Medical University of Warsaw, Warsaw, Poland

**Keywords:** Kager fat pad, Achilles tendon, Retrocalcaneal bursa, Crural fascia

## Abstract

**Purpose:**

The aim of the study was to map connections within the Kager’s fat pad between the structures which limit it.

**Methods:**

A retrospective re-review of 200 ankle magnetic resonance imaging (MRI) examination was conducted. Connections within the Kager’s fat pad between the superior peroneal retinaculum, the fibulotalocalcaneal ligament, the posterior talocalcaneal ligament, the flexor hallucis longus, the paratenon of the Achilles tendon, the flexor retinaculum and bones were studied and a model of the connections was constructed.

**Results:**

The superior peroneal retinaculum was directly connected with the fibulotalocalcaneal ligament in 85.5% of cases, the lateral part of the paratenon in 82.5%, the processus posterior tali in 78.5%, the posterior talofibular ligament in 32%, the flexor retinaculum in 29.5% and the anterior talofibular ligament in 9%.

The fibulotalocalcaneal ligament was connected with the paratenon (on the medial side 88.5%, on the lateral side 68.5%), the flexor retinaculum in 70%, the posterior process of the talus in 79%, the osteofibrosus tunnel for the flexor hallucis longus in 53%, the posterior talofibular ligament in 43.5% and the calcaneofibular ligament in 10.5%.

The posterior talocalcaneal ligament was connected with the fibulotalocalcaneal ligament in 71%, with the osteofibrosus tunnel for the flexor hallucis longus in 76.5%, with the flexor retinaculum in 70%. The plantaris tendon showed projection to the crural fascia in 34 of % cases.

**Conclusion:**

In the Kager’s fat pad there are present more connections than previously reported. All the connections unit at the level of the posterior process of the talus.

## Introduction

Connections within the Kager’s fat pad (KFP) have not been comprehensively studied. The KFP is an adipose structure located between the Achilles tendon, the flexor hallucis longus (FHL) and the calcaneus [8, 28]. Due to the KFP’s close relation to the Achilles tendon, it can be involved in traumatic changes in the tendon’s midportion as well as the entheses or other conditions like a tumor or infection [11, 22, 26], but the connections can provide stability [5, 10]. The relatively large size of the KFP enables its individual parts to have different functions and exhibit slightly different mechanical functions and a slightly different sort of pathology [3, 15, 28]. The wedge-shaped part of the KFP participates in the lubrication of the anterior outline and distal part of the Achilles tendon and evenly distributes stress at the Achilles enthesis, and removes debris from the retrocalcaneal bursa [2, 22]. The KFP also has fibrous bands stretching between the surrounding structures in a diversified distribution. Different parts of the KFP may play different functions. In previous studies, only two connections have been found within the KFP: the posterior talocalcaneal ligament (PTCL) and the fibulotalocalcaneal ligament (FTCL) [6, 19]. The PTCL originates on the lateral tubercle on the posterior process of the talus and inserts on the superior outline of the calcaneus [6]. The FTCL originates in the anterior part of the malleolar groove, runs medially, then divides into flat sheet-like laminas, the superomedial and the inferolateral. The first one inserts onto the lateral tubercle while the second one inserts onto the superior outline of the calcaneus [19]. It should be noted that there is an inconsequence in the nomenclature of the FTCL’s laminas. The one which inserts onto talus is sometimes called horizontal or talar, while the one which runs toward the calcaneus, inferolateral or peroneocalcaneal [20].

The plantaris tendon may have an insertion into the deep crural fascia which corresponds with type 4 according to Olewik et al. (2016) [18]. It was previously mentioned that the plantaris tendon may have a connection to the flexor retinaculum or to the crural fascia [1, 27].

MRI is an excellent method that allows tissue differentiation with high resolution [3, 7]. Thanks to this, the presence of even fine fiber bands within the KFP can be precisely demonstrated [21, 23].

Despite the common occurrence of soft tissue alterations in the KFP [3], to our knowledge, there is a lack of modern radiological research on a large amount of material describing and classifying the presence of fibrous connections and ligaments in the KFP.

The purpose of this study was to examine the occurrence and variability of bands and ligaments in the KFP and construct a map of the connections based on MRI.

## Materials and methods

We conducted a retrospective re-evaluation of 200 ankle MRI of athletes (112 males and 88 females; age range 16–47 years, mean 29 years; 101 right ankles and 99 left ones). Exclusion criteria were the presence of the os trigonum (21 cases), a history of previous fracture and obvious abnormality within the KFP because of the possibility of a focal reaction and finally remaining orthopedic hardware due to the possible artifacts (16 cases). Totally 200 patients with ankle MRI fulfilling inclusion and exclusion criteria were included in our study. MRI examinations were performed at our institution over six months (between November 2018 and April 2019), about 2–4 weeks after the ankle injury (mean duration 2.8 weeks) for clinical purposes. All examinations were performed using MRI machine Ingenia 3.0 T MR system (Philips Healthcare) with a dedicated ankle coil.

To obtain the best possible differentiation of fat and fibrous tissue, we used sequences without fat tissue saturation in PD (proton density) and T2-weighted images in three different planes. The sagittal and axial FSE (fast spin-echo) sequences had a field of view (FOV) of 14 × 14 cm, a slice thickness of 3 mm without spacing. The coronal sequences had a FOV of 10 × 8 cm. Matrix in axial plane 256 × 218, in coronal plane 256 × 230 in sagittal plane 320 × 272. The echo time in PD 20 while in T2 FSE 4000 ms.

In the study, we studied connections between the following structures: the peroneal tendons and superior peroneal retinaculum, the flexor hallucis longus, the paratenon, the medial vascular space, the tibialis posterior and the flexor digitorum longus. The hypothesis of our study was that located within the KFP are fascia projections joining the different limitations of the KFP.

After coding the results in the table, a model of connections developed in the KFP at the level of the posterior process of the talus was made. Due to earlier reports regarding the plantaris tendon and its relationship to the fascia, its variant according to Olewik et al. [18] was applied.

## Results

In the KFP a lot more connections than only the FTCL or the posterior talofibular ligament (PTFL) can be found as was reported in previous studies. Most of the connections we have found are projections of the crural fascia orientated in the frontal plane, therefore their assessment in the horizontal plane is the most optimal. Due to the very narrow anterior–posterior diameter, assessment in the frontal plane is more difficult. The sagittal plane helps to assess the cranio-caudal distension of the described connections.

**Fig. 1:**
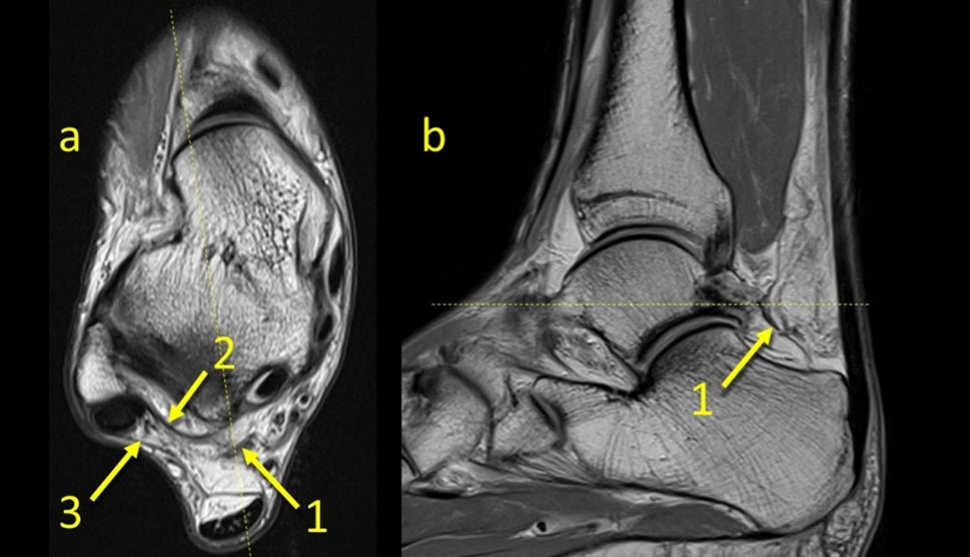
The posterior talocalcaneal ligament. **1** – the FTCL, **2** – the superomedial lamina of the FTCL, **3** – the superior peroneal retinaculum

**Fig. 2:**
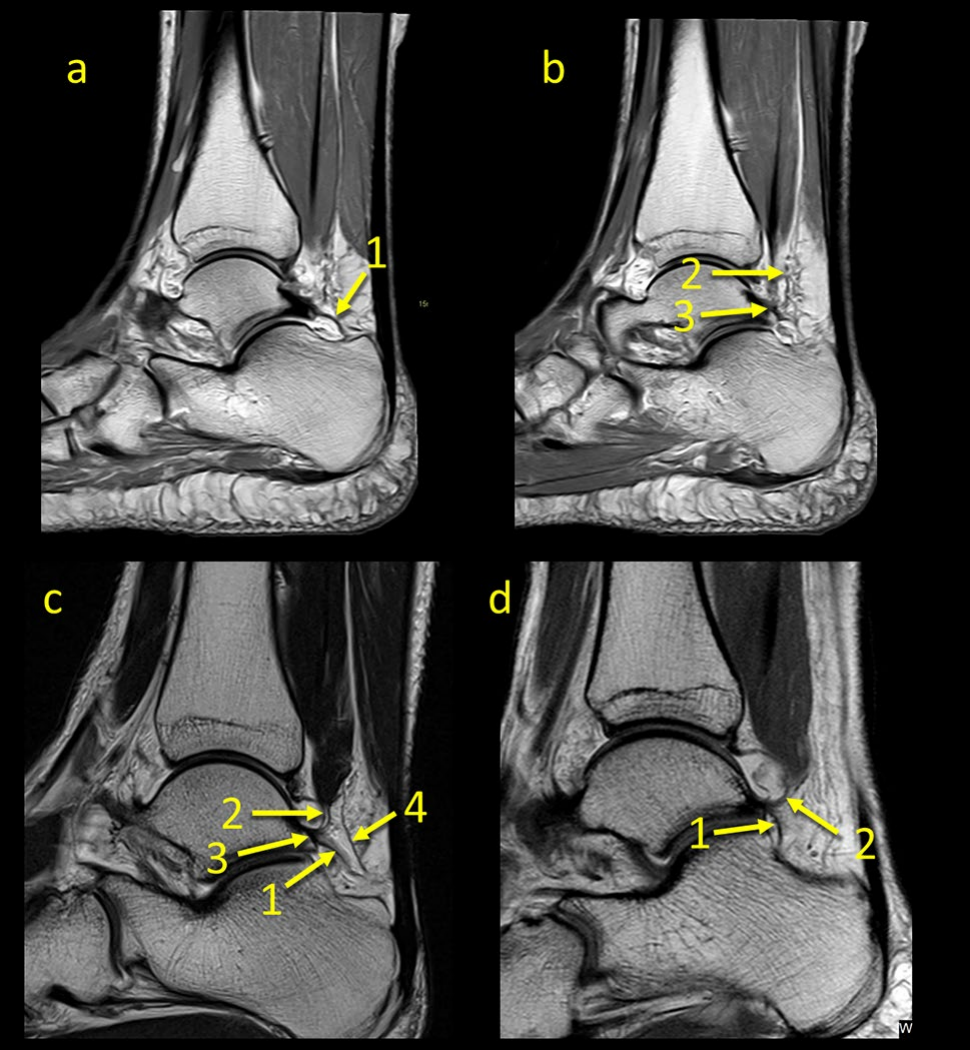
The posterior talocalcaneal ligaments connections in two different patients, a and b, c and d. **1** – the PTCL, **2** – the superomedial lamina of the FTCL connecting the FHL, **3** – the superomedial lamina of the FTCL’s insertion on the posterior process of the talus, **4** – the communication between the PTCL and the FHL

All connections could be identified at the level of the posterior process of the talus on the axial plane. The PTCL was identified in *n* = 161 (80.5%) (Figs. 1, 2, 6, Table 1). This ligament originates from the lateral tubercle of the posterior process of the talus where it connects with the superomedial lamina of the FTCL and then descends to the superior outline of the calcaneal tuber (Fig. 6). In *n* = 153 (76.5%) of cases, its vertical projection was connected directly to the FHL (Figs. 2c, 6), and indirectly via the FTCL in 39 cases (19.5%) (Fig. 2b–d).

**Table 1:** Number of direct connections between the structures include in the study, % in brackets, *n* = 200 (100%)

	SPR	ATFL	CFL	PTFL	FTCL	PTCL	PAT med	PAT lat	PPT	OF-FHL	FR
SPR	x	18 (9)	0 (0)	64 (32)	171 (85.5)	17 (8.5)	39 (19.5)	165 (82.5)	157 (78.5)	0 (0)	59 (29.5)
ATFL		x	ns	ns	0 (0)	0 (0)	0 (0)	0 (0)	0 (0)	0 (0)	0 (0)
CFL			x	ns	21 (10.5)	0 (0)	0 (0)	0 (0)	0 (0)	0 (0)	0 (0)
PTFL				x	87 (43.5)	0 (0)	0 (0)	0 (0)	ins	0 (0)	0 (0)
FTCL					x	142 (71)*, 133 (66.5)**	177 (88.5)	137 (68.5)	158 (79) ***	105 (52.5)	140 (70)
PTCL						x	16 (8)	24 (12)	orig	153 (76.5)	36 (18)
PAR med							x	****	0 (0)	0 (0)	117 (58.5)
PAR lat								x	0 (0)	0 (0)	0 (0)
PPT									x	ins	0 (0)
FHL										x	0 (0)
FR											X

*ins.* insertion, *FR* the flexor retinaculum, *ns* not studied, *OF-FHL* osteofibrous tunnel for FHL, orig.-origin, *SPR* the superior peroneal retinaculum, *PAT med* the medial part of the paratenon, *PAT lat.* the lateral part of the paratenon,, *PPT* the posterior process of talus

*The superomedial lamina of FTCL

**Inferolateral lamina of the FTCL

***Only variant one of the FTCL

****Anatomical continuity

The superior peroneal retinaculum’s connections were observed to be variable (Fig. 10, Table 1). The connections most often seen were with the FTCL, the lateral part of the paratenon of the Achilles tendon and the posterior process of the talus (Table 1, Fig. 2). Connections with the lateral ankle ligament complex or the flexor retinaculum are visible but much less so.

**Fig. 3:**
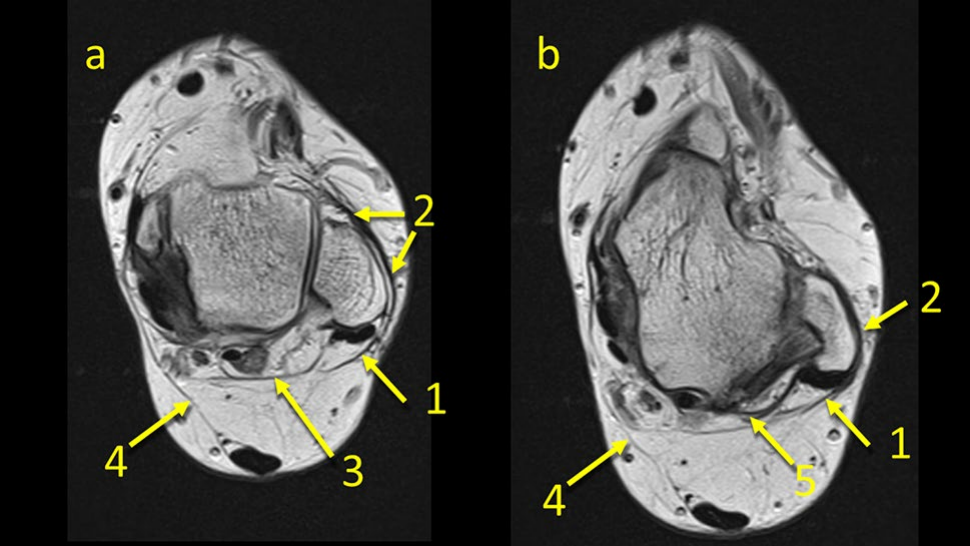
The communication between the ATFL and the superior peroneal retinaculum. **1** – the superior peroneal retinaculum, **2** – the fibers connecting the superior peroneal retinaculum with the ATFL, **3** – the FTCL, **4** – the communication between the flexor retinaculum and the medial paratenon, **5** – the superomedial lamina of the FTCL

**Fig. 4:**
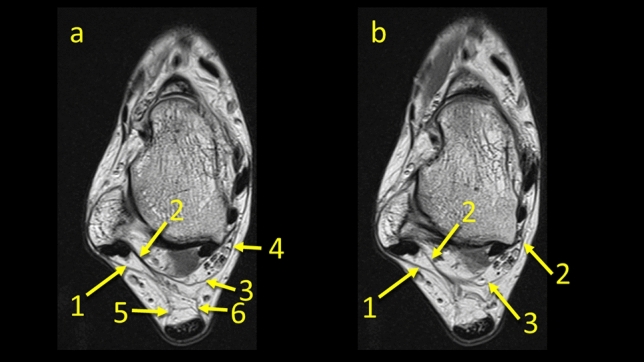
The communication between the superior peroneal retinaculum and the flexor retinaculum. **1** – the superior peroneal retinaculum, **2** – the FTCL, **3** – the communication between the superior peroneal retinaculum and the flexor retinaculum via the FTCL, **4** – the flexor retinaculum, **5** – the medial part of paratenon, **6** – the lateral part of paratenon

**Fig. 5:**
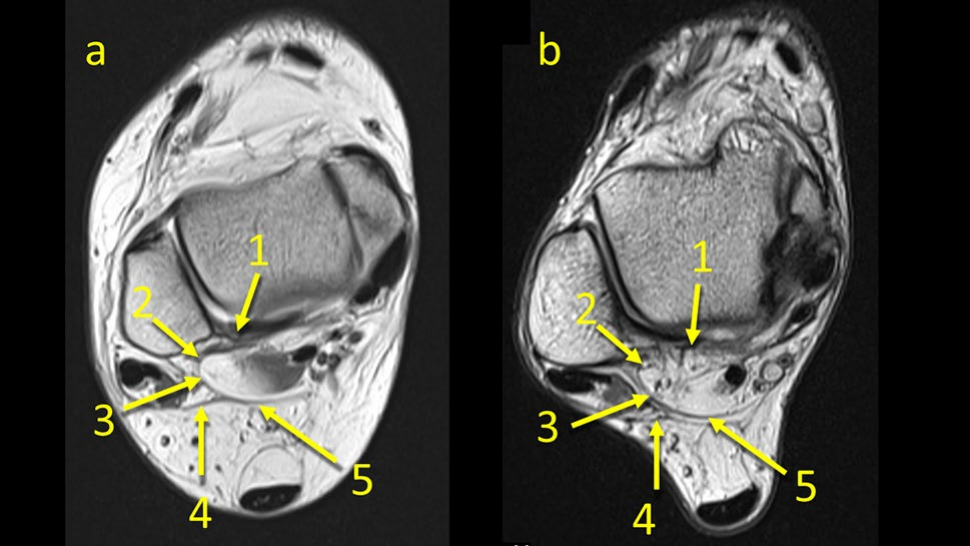
Two patients with communication between the FTCL and the PTFL (the arrow number two). **1** – the PTFL, **2** – the communication, **3** – the FTCL, **4** – the superior peroneal retinaculum, **5** – the superomedial lamina of the FTCL

**Fig. 6:**
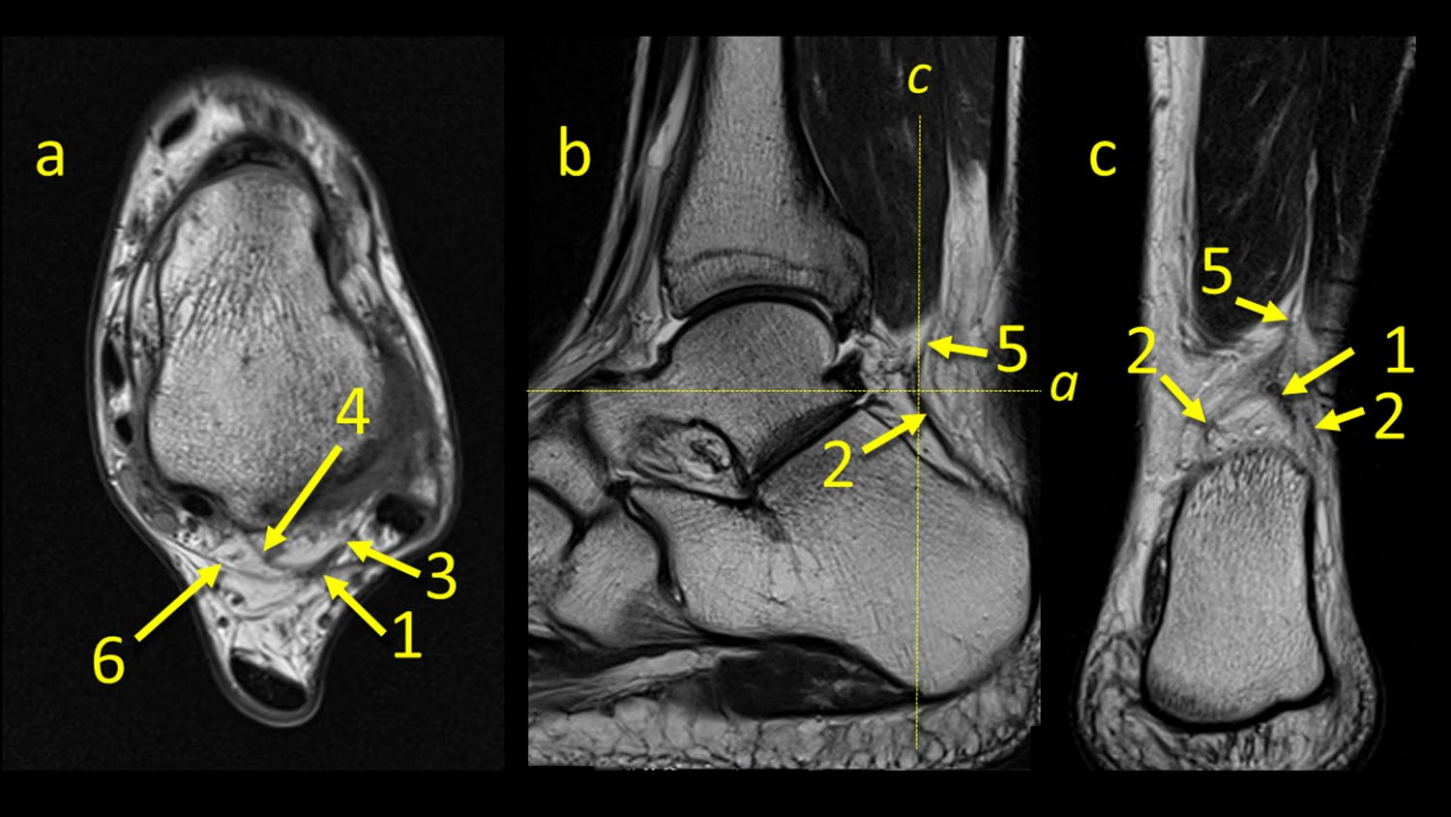
The node-like structure between ligaments. **1** – the node-like structure, **2** – the PTCL, **3** – the FTCL, **4** – the superomedial lamina of the FTCL, **5** – the connection with the FHL, **6** – the connection to the flexor retinaculum

In *n* = 18 cases (9%), communication to the anterior talofibular ligament (ATFL) was found as prolongation or extension of the superior peroneal retinaculum fibers over the external outline of the lateral malleolus to the superior part of the ATFL (Figs. 3, 4, 5, 6).

**Fig. 7:**
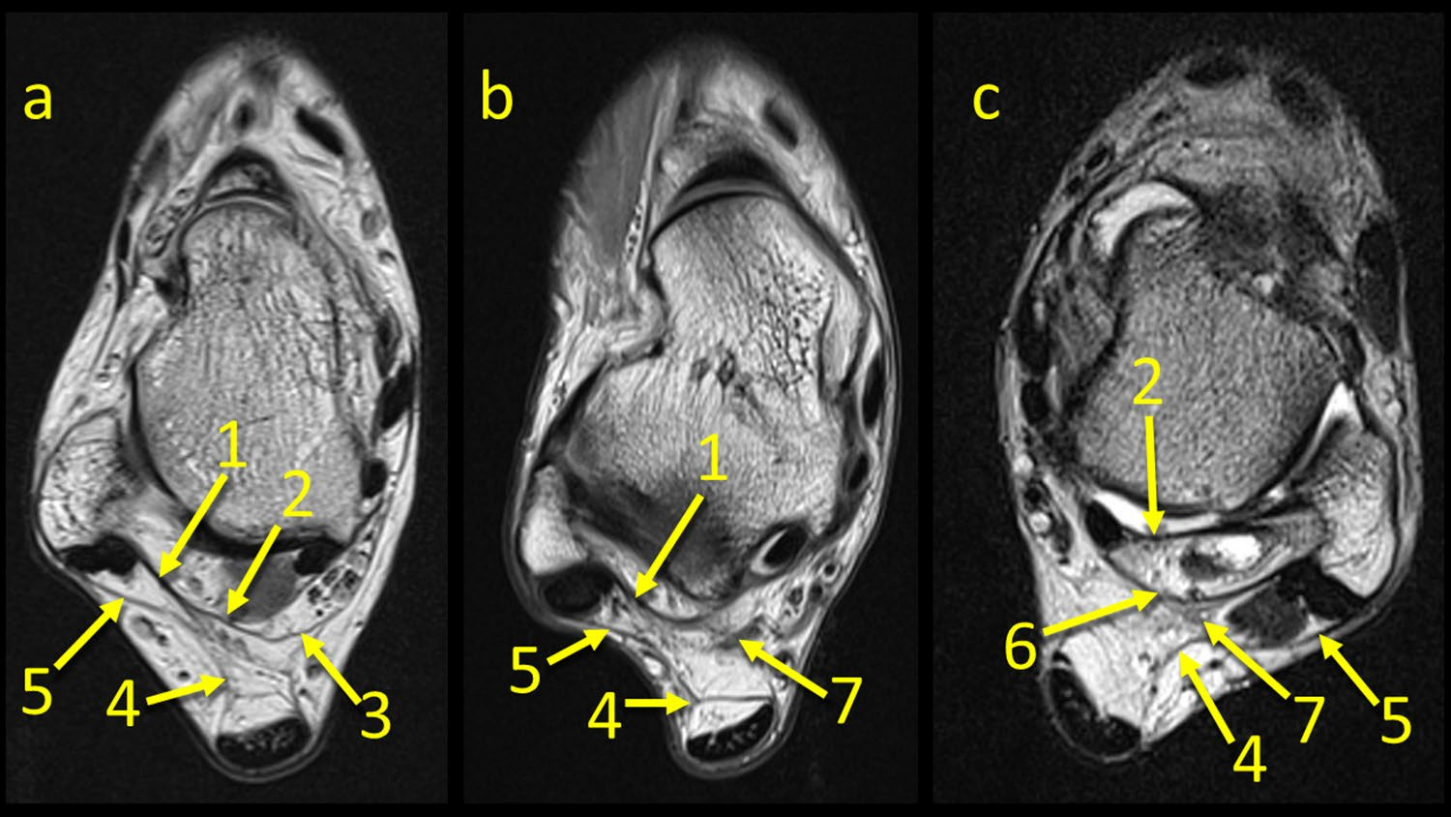
Three variants of the FTCL. **1** – the FTCL, **2** – the superomedial lamina of the FTCL**, 3** – the communication between the superior peroneal retinaculum and the flexor retinaculum via the FTCL, **4** – the lateral part of the paratenon, **5** – the superior peroneal retinaculum, **6** – the inferolateral lamina of the FTCL, **7** – the “node-like” structure

The FTCL is a structure which is located between the lateral malleolus and the medial vascular space at the level of the posterior process of the talus. It was present in all cases in two variants either as two laminas, type 1 (*n* = 158, 79%) or as a single structure, type 2 (*n* = 42, 21%), Fig. 7. Its origin is located in the fossa malleoli lateralis, then runs medially where in type 1 it branches into the superomedial and inferolateral lamina. In type 2, the superomedial lamina is not present. This ligament, thanks to its central localization and connections, serves as a “common intersection points” between the structures which limit the KFP (Table 1). In some cases, *n* = 22 (11%), division of the FTCL is already seen at its origin on the fibula, making the superomedial and inferolateral laminas longer than usual (Fig. 7c). The superomedial lamina inserts into the lateral tubercle of the processus posterior tali, contributing to the osseofibrous tunnel for the FHL, while the inferolateral lamina which runs inferiorly and inserts onto the calcaneus.

The most constant FTCL connection was with the paratenon of the Achilles tendon. The connection with the flexor retinaculum was the most medially located connection, while that with the FHL is the most superior connection (Table 1).

At FTCL’s origin in the fossa malleoli lateralis, the connection with the PTFL was seen in *n* = 87 cases (43.5%) (Fig. 5), while the connection with the calcaneofibular ligament (CFL) was identified in *n* = 21 cases (10.5%) (Table 1). In no cases was direct contact with the AFTL recognized. We noticed the FTCL’s connections to the FHL in 105 cases 52.5% of cases (Fig. 2).

The plantaris tendon in type 4 according to Olewnik et al. [18], *n* = 67 (34%), showed a projection of its insertion to the crural fascia. The plantaris tendon was located medially to the Achilles tendon in direct contact with the medial part of the paratenon. The accessory slip from the flexor retinaculum with the medial part of the paratenon was noticed in *n* = 77 cases (38.5%). We found one case of the peroneus quartus tendon but the connections within the KFP did not differ from other cases. No variations of the solus tendon were identified.

The paratenon of the Achilles tendon was connected anteriorly, medially and laterally with the FTCL (Table 1) and with the superior peroneal retinaculum. Anteriorly and medially connection to the node-like structure can be seen.

The lateral tubercle of the posterior process of the talus is the site of insertion of the PTCL, the superomedial lamina of the FTCL and part of the PTFL. The prolongation of the superomedial lamina of the FTCL continues to the medial tubercle, limiting the osteofibrous tunnel for the FHL, and finally joins with the flexor retinaculum. Often the septum between the flexor digitorum longus and the tibialis posterior tendon is visible (*n* = 157 cases, 79%).

## Discussion

In our study, we found many more connections than the two previously described ligaments. To our knowledge, there are no radiological studies regarding all connections within the KFP. In some earlier published studies only on cadavers, only information about the posterior talocalcaneal ligament and the FTCL can be found [13, 20]. However, when assessing ankle MRI, many more connections within the KFP can be observed. Our study revealed the presence of variable connections and some “common intersection points” between the fascial projections or ligaments within the KFP. The most classic example is the occurrence of a node-like structure located in the central part of the KFP at the level of the posterior process of the talus, as part of the FTCL. Due to the small amount of research, unambiguous nomenclature for fascial connections is missing [19, 20]. The connections revealed in our study are part of the fascial system proposed previously [25], but because of the complexity of further development in fascial nomenclature is required.

The occurrence of such connecting structures confirms the theories of occurrence of the intermuscular myofascial connections which can influence muscle and tendon functions [12]. A fascia may have passive transmitting mechanical forces generated by skeletal muscle. Our study revealed the crural fascia connections to tendons, e.g., to the FHL or to the paratenon. The plantaris tendon’s connection to the crural fascia and its projections to the paratenon may partially explain the anatomical basis of the pathology of Achilles tendon tendinopathy and paratenonitis [18]. Some authors reveal that the connection of the KFP to the Achilles tendon helps to maintain its stability [5, 10]. The connections of the KFP with the paratenon revealed in our study can be an anatomical background which may decrease the mutual glide of the paratenon and the Achilles tendon. This reduction can develop of the overuse changes, the paratendinitis or the tendinopathy of the Achilles tendon.

The presence of connections to ligaments such as the PTFL or CFL helps to understand why isolated tears are rare in the ankle area [9, 17]. Given the existence of the connections within the KFP, it is somewhat more logical to understand the coexistence of the overuse of the Achilles tendon with alterations in other structures in the ankle. We believe, therefore, that injuries to the ankle area should be considered holistically, taking into account the mechanism of the injury and not just one individual injured structure.

Several connections revealed in the posterior ankle area look like the connections described earlier in the lateral ankle complex [9, 17, 30]. Furthermore, there are some similarities between the KFP and the Hoffa fat pad [14]. Alterations in these structures result in the alteration of the KFP’s structure and obscure its outline on radiological imaging which is known as the Kager’s fad pad sign [10]. Impingement, inflammation, overuse or infection in the KFP may finally lead to the lipomatous tissue necrosis [4, 22, 29]. The KFP shows alterations in the case of the Haglund deformity because of the impingement and fat edema, synovitis in the deep calcaneal bursa and a high signal in the Achilles tendon insertion [3, 16, 30]. However, no connections were revealed in this part of the KFP which allows it to move freely in the retrocalcaneal bursa [8, 28]. We revealed that the KFP is connected with paratenon on both the medial and lateral sides which may explain the KFP’s involvement in the pathology of the enthesis organ of the Achilles tenon or paratenon [22, 24, 26].

**Fig. 8:**
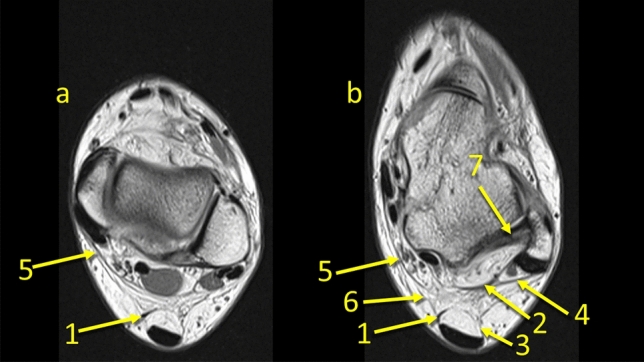
The connection between the paratenon and the superior peroneal retinaculum and the flexor retinaculum. **1** – the plantaris tendon, **2** – the FTCL, **3** – the lateral part of paratenon, **4** – the superior peroneal retinaculum, **5** – the flexor retinaculum, **6** – the connection between the paratenon and the flexor retinaculum, **7** – the PTFL

**Fig. 9:**
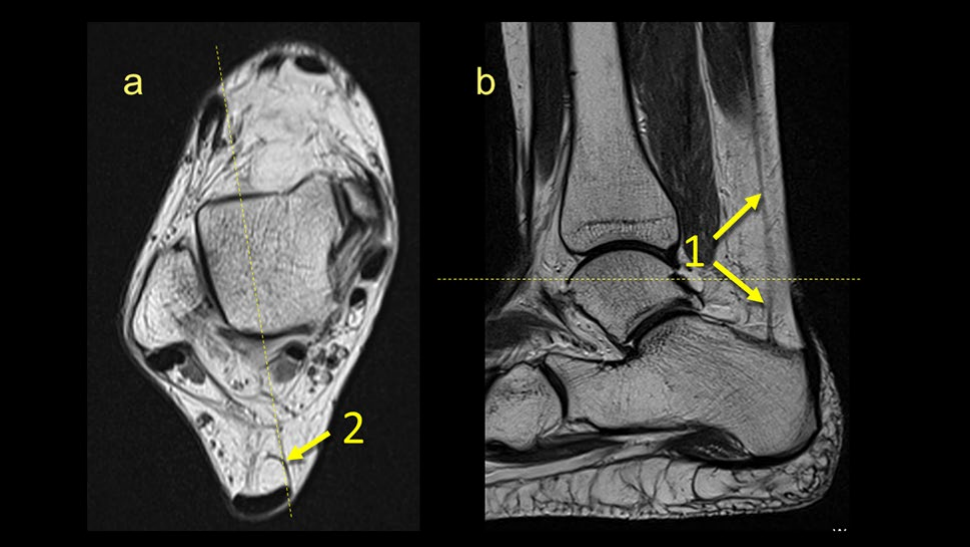
The connection between the plantaris tendon and the paratenon. **1** – the plantaris tenon, **2** – the plantaris inserts into the medial part of the paratenon

**Fig. 10:**
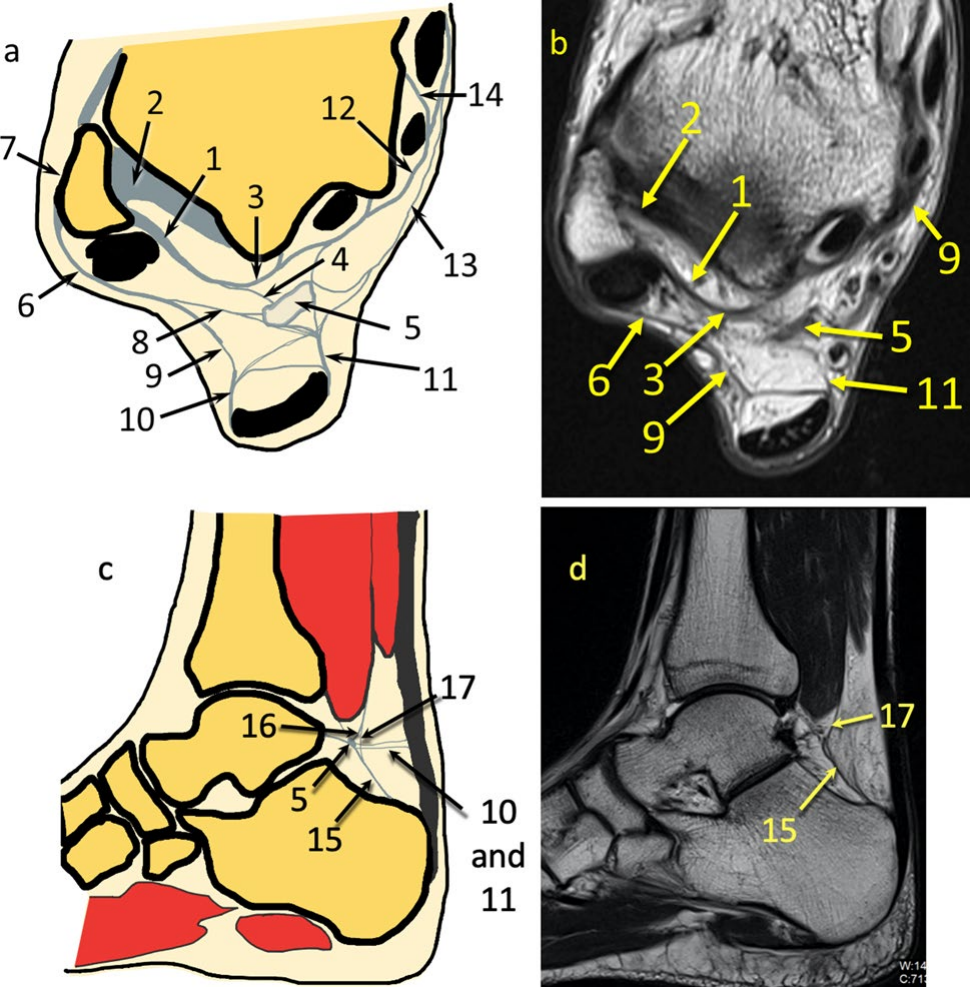
Communications within the KFP at the level of the posterior process of the talus. **a** and **c**- general map of the communications between different fibrotic structures, **b** and **d**- example of the patient. **1** – the FTCL, **2**- the PTFL, **3**- the superomedial lamina of the TFCL, **4**- branch of the TFCL to the node-like structure, **5**- the node-like structures, **6**- the superior peroneal retinaculum, **7**- connection between the ATFL and the superior peroneal retinaculum, **8**- connection between the node-like structure and the superior peroneal retinaculum, **9**- connection between the lateral part of the paratenon (**10**) and the superior peroneal retinaculum, **11**- connection between the node-like structure and the medial part of the retinaculum, **12**- the flexor retinaculum, **13**- connection between the node-like structure and the flexor retinaculum, **14**- the septa between the flexor digitorum longus and the tibialis posterior, **15** – the PTCL, **16** – connection between the PTCL and FHL via the node-like structure, **17** – direct connection between the PTCL and FHL

The posterior process of the talus is a point of insertion of the superomedial lamina of the FTCL which is a connection between the lateral side of the ankle by means of the PTFL [19, 20]. This is the reason why we decided to evaluate the KFP’s connections at this level. Medially, the FTCL runs and connects with the flexor retinaculum. Inferiorly, it connects with the posterior talocalcaneal ligament. Previously published results indicate that the inferolateral lamina inserts on the lateral part of the tuber calcanei, but in our study, we found that this arch-shaped structure inserts on the superior surface of the calcaneus (Fig. 6c). The discrepancy may be due to the fact that previous work was based on scarce material, for example Peduto et al. dissected six cadavers [20] and Pastore et al. dissected ten cadavers [19] (Fig. 8, 9, 10).

Our study showed that the PTCL was found in about 70% which is similar to number reported by Pastore et al. [19], while a recently published study by Iovane et al. [6] indicate that the incidence of the PTCL is much lower, estimated at nearly 9%. It is difficult to determine where these discrepancies come from, possibly a slightly different research methodology or a different selection of material.

In some cases, the plantaris tendon attaches to the fascial structures at the site where the flexor retinaculum meets the medial part of the paratenon of the Achilles tendon. Under adverse conditions, the plantaris tendon may have an important role in triggering Achilles tendonitis or overuse [18]. As in the case of the palmaris longus tendon connecting with the palmar fascia, the plantaris tendon connects with the crural fascia and may have a connection with the plantar fascia. This fascial connection was previously described but in our study.

We reveal that this connection extends further to other structures making the KFP a connecting, not a separating structure. Considering previously published results of functional studies approach to the KFP’s function should be revised [15].

There are some limitations to this study. Due to its retrospective character, we had no influence on the thickness of the layers in the MRI examination because the examinations were performed because of clinical indications, in a defined protocol. Another limitation is the lack of surgical-anatomical correlation and the presence of discrete degenerative changes.

## Conclusion

There are significantly more connections in KFP than previously reported. The KFP is a structure where the crural fascia’s projections from the KFP limiting's structures join. The most important “coordination structure” is located at the level of the posterior process of the talus where the following unite: the PTCL, the FTCL and its projections to the superior peroneal retinaculum, the flexor retinaculum joint, the paratenon of the Achilles tendon and the FHL. The connections shown in our study indicate the presence of a fascial integration system within the KFP. The current study revealed that the KFP can play an important role in the pathology of its limiting structures. One structure’s dysfunction due to connections may cause the dysfunction of another. The existence of these connections may partly explain the multifactorial cause of Achilles tendinopathy.

## Abbreviations

ATFL: The anterior talofibular ligament
CFL: Calcaneofibular ligament
FHL: The flexor hallucis longus
FOV: Field of view
FSE: Fast spin-echo
FTCL: The fibulotalocalcaneal ligament
KFP: The Kager’s fat pad
MRI: Magnetic resonance imaging
PD: Proton density
PTCL: The posterior talocalcaneal ligament
PTFL: The posterior talofibular ligament
TE: Echo time
